# The Association Between the Use of Oclacitinib and Antibacterial Therapy in Dogs With Allergic Dermatitis: A Retrospective Case-Control Study

**DOI:** 10.3389/fvets.2021.631443

**Published:** 2021-02-15

**Authors:** Hester Rynhoud, Justine S. Gibson, Erika Meler, Ricardo J. Soares Magalhães

**Affiliations:** ^1^UQ Spatial Epidemiology Laboratory, School of Veterinary Science, The University of Queensland, Gatton, QLD, Australia; ^2^School of Veterinary Science, The University of Queensland, Gatton, QLD, Australia; ^3^Children Health and Environment Program, Child Health Research Centre, The University of Queensland, South Brisbane, QLD, Australia

**Keywords:** oclacitinib, allergic dermatitis, antibacterial, anti-pruritic, dogs

## Abstract

**Background:** Canine allergic dermatitis, including atopic dermatitis, often requires antibacterial therapy for concurrent infections. Oclacitinib is indicated for treatment of pruritus associated with allergic dermatitis and the clinical manifestations of atopic dermatitis in dogs aged ≥12 months.

**Hypothesis/Objectives:** We aimed to determine if there was a quantitative difference in antibacterial use by dogs with allergic dermatitis receiving oclacitinib vs. other anti-pruritic therapies and before vs. after oclacitinib.

**Animals:** In this retrospective case-control study, cases (*n* = 58) included dogs suffering from allergic dermatitis aged ≥12 months receiving oclacitinib and controls (*n* = 205) were counterpart dogs treated with other anti-pruritic therapies.

**Methods:** Clinical histories of dogs with allergic dermatitis were collected from a small animal university hospital. Multivariable logistic regression models were developed adjusting for underlying skin or ear conditions to determine whether cases were prescribed fewer antibacterials than controls.

**Results:** The odds of systemic antibacterial usage were lower in cases vs. controls [odds ratio (OR): 0.29 (95% confidence interval 0.12–0.71); *P* = 0.007]. The odds of amoxycillin clavulanic acid usage (12.5–25 mg/kg orally every 12 h) was lower in cases vs. controls [OR: 0.08 (0.01–0.71); *P* = 0.024]. Topical antibacterial drug use was reduced overall; however, only the odds of neomycin use was lower in cases vs. controls [OR: 0.3 (0.1–0.89); *P* = 0.029]. Cases had higher odds of experiencing improvements in allergic dermatitis categories vs. controls [OR: 7.89 (3.26–19.13); *P* < 0.001].

**Conclusions and Clinical Importance:** Our results suggest that use of oclacitinib to treat allergic dermatitis in dogs is associated with less antibacterial use than other anti-pruritic therapies.

## Introduction

Dogs with allergic dermatitis, including atopic dermatitis, contact allergy, flea allergy dermatitis, and cutaneous adverse food reactions, present to veterinary clinics with the characteristic clinical sign of pruritus which is often associated with gross inflammation and secondary skin infections (1). Environmental triggers for acute flares of atopic dermatitis can include food, pollens, dust mites, and flea or other insect bites (2). Atopic dermatitis is a common allergic skin disease of dogs which can negatively influence pet and owner quality of life; therefore, effective treatment is necessary (3–6).

Treatment of allergic dermatitis aims to control pruritus and inflammation, as well as identify and eliminate underlying etiologies to prevent and reduce acute flares (2). Topical and systemic glucocorticoids can be used to treat both acute flares and chronic allergic dermatitis, respectively (2). Long-term glucocorticoid therapy is no longer recommended due to serious adverse effects, including gastrointestinal ulceration, hyperlipidaemia, diabetes mellitus, muscle wasting and iatrogenic hyperadrenocorticism (7). Systemic cyclosporin and topical tacrolimus can also be used to manage atopic dermatitis; however, the slow onset of action renders them inappropriate for acute flares (2). Lokivetmab, is also available to treat atopic dermatitis which has recently been reported to have a more pronounced effect on pruritis compared to cyclosporin (8). Antihistamines are also often used but are of limited to no benefit for dogs with atopic dermatitis (2). In addition, the use of non-irritating shampoos and oral essential fatty acid supplements has been suggested to support skin barrier function (2, 9). Alleviating pruritus is important when treating allergic dermatitis to interrupt the itch cycle and allow for skin healing, thus reducing chronic inflammatory changes and secondary infections (10).

Dogs with allergic dermatitis frequently have concurrent infections of the skin and ears (9, 11). Atopic dogs have a higher abundance of *Staphylococcus* spp. on their skin, notably *Staphylococcus pseudintermedius*, which is commonly implicated in pyoderma (12–14). Studies infer that staphylococci have increased adherence to inflamed and atopic skin, which could explain the increased abundance of this organism (15, 16). It has been suggested that these bacteria are also involved in hypersensitivity responses, most commonly due to staphylococcal components or toxins acting as superantigens (13). Treatment often requires topical and/or systemic antibacterial therapy (11). With increasing reports of bacterial resistance to antimicrobial therapy in humans and animals, veterinarians are encouraged to follow antimicrobial stewardship (AMS) guidelines when treating these cases to improve efficacy and limit further development of resistance (2, 17).

One way to aid AMS is to use treatments that reduce the need for antibacterials when treating allergic skin disease. Oclacitinib, a Janus kinase (JAK) inhibitor, targets specific pathways for cytokines involved in itch and inflammation (18). The JAK 1 enzyme is involved in signaling and signal transduction of pro-inflammatory, pro-allergic and pruritogenic cytokines associated with atopic dermatitis (10). Oclacitinib inhibits predominantly JAK 1 dependent cytokines and effectively treats the clinical signs associated with allergic dermatitis and atopic dermatitis in dogs (18).

The primary aim of this retrospective study was to quantify changes in systemic and topical antibacterial use in client-owned dogs with allergic dermatitis, including atopic dermatitis, following administration of oclacitinib compared to dogs that were not prescribed oclacitinib, and also before and after initial oclacitinib use. The effects of oclacitinib on corticosteroid use and skin conditions were secondary objectives.

## Materials and Methods

### Ethical Statement

The data used were originally collected by veterinarians undertaking standard veterinary practices, therefore animal ethics approval was not required. Animals were deidentified during the data collection process for this study and data was stored and analyzed following the University of Queensland's research integrity protocols.

### Case Definitions and Data Sources

All dogs included in the retrospective case-control study were ≥12 months old in accordance with the minimum approved age for administration of oclacitinib. Cases and controls were initially identified using specific search terms as listed in Supplementary Table 1. Only on label conditions were included in the initial search terms. Dogs were conditionally diagnosed with an allergic skin condition if a definitive diagnoses was not provided. Diagnostic tests performed to rule out other causes of itchy skin included ear smears, flea combs, skin scrapes, sticky tape, and cultures; however, not all consultation notes had record of these tests.

Inclusion criteria for cases included dogs diagnosed with an allergic skin condition (atopic dermatitis, contact allergy, flea allergy dermatitis, cutaneous adverse food reactions) that were treated with oclacitinib. Cases were identified in the veterinary clinical database between January 2016 and July 2018 in accordance with oclacitinib's release to market in Australia in early 2016. Medical history was collected from the date of initial diagnosis with an allergic skin condition. In total, consultation data for cases were sourced between November 2010 and July 2018, allowing case data to be classified into “before oclacitinib” and “after oclacitinib.”

Inclusion criteria for controls included dogs diagnosed with an allergic skin condition that were not treated with oclacitinib. Allergic dermatitis, including atopic dermatitis, is often managed multimodally, therefore other therapeutics were frequently used in the management of these dogs. Controls were identified in the clinical database as dogs that never received oclacitinib, with data collected from clinic consultations between January 2010 and July 2018. The consultations included in the analysis also counted medication dispensations (where consultation notes only included medication dispensations for both cases and controls).

Data were extracted from the veterinary clinical database at a university small animal teaching hospital. Demographic data collected included signalment, breed, size, and reproductive status. Dogs were grouped in categories according to size as small, medium or large, based on Pickup et al. (19) who used these categories according to the UK Kennel Club classification (20). Clinical history data included consultation type, date, clinical signs, diagnostic tests, diagnosis, treatment plans, drugs administered, dosage and dose frequencies and outcome of most recent consultation. Antibacterials were divided into topical and systemic which included agents used for cutaneous and otic conditions. Antibacterials included antiseptics and biocides. Every time a drug, including oclacitinib, was prescribed and dispensed in a consultation this was defined as a “course.” A detailed description of the data used is available in Supplementary Table 2.

### Statistical Analyses

#### Descriptive Analysis

Statistical analyses involved comparisons between case (dogs treated with oclacitinib) and control data and within case data before and after initial oclacitinib use. Topical and systemic antibacterial usage was depicted as total number of courses prescribed per dog for all consultations recorded in the study. All statistical analyses comparing cases and controls (including regression models) used case data post-oclacitinib. Pre-oclacitinib data was only used when comparing before vs. after oclacitinib use within cases. The total timespan for the cases both pre- and post-oclacitinib use was November 2011 to July 2018. The timespan for cases post-oclacitinib use was from March 2016 to July 2018. The distribution of the number of antibacterial courses per dog was not normally distributed and so non-parametric tests were used. To detect whether the use of topical and systemic antibacterials differed significantly between cases and controls, a Wilcoxon rank-sum test was conducted. To determine whether there was a significant difference in topical and systemic antibacterial use before and after initial oclacitinib use in cases, a Wilcoxon signed-rank test was performed. Similar tests were performed to determine differences between total number of courses per dog in small, medium and large breeds. To determine if the number of courses of oclacitinib influenced antimicrobial use within the cases, a Kruskal–Wallis test was performed to identify whether there were significant differences in antimicrobial use within cases based on the number of oclacitinib courses they received (one course, two courses, or three or more courses). A chi-square test was used to determine if the proportional use of systemic and topical antibacterials differed significantly between the cases and controls and before and after oclacitinib use in cases. All descriptive statistical analyses were performed using Microsoft Excel 2016 and Stata version 13.1 (Stata Corporation, College Station, TX, USA).

#### Models Adjusted for Overall Drug Use and Specific Drug Groups

Bernoulli logistic regression models were developed with oclacitinib treated dogs (cases) as the outcome of interest and dogs not treated with oclacitinib as the reference group; this approach models the probability of being a case. The predictor variables used in the logistic regression models are listed in Supplementary Table 3. Analysis was conducted in two phases. First, the univariable association between cases and different drug therapies was evaluated using univariable logistic regression. A cut off *P*-value of ≤0.20 was used when selecting predictor variables to be included in the full multivariable model. Pearson's correlation coefficient was used to test for correlation between variables with a *P*-value ≤0.20. If a pair of variables had a correlation coefficient >0.8 then only one was considered in the full multivariable model. Second, variables that were significant in the univariable analysis were included in a full multivariable model. The final multivariable model was arrived at using a manual backward stepwise variable selection process. Confounding variables were identified by assessing the impact of variable removal on the coefficients of the remaining variables. If the coefficient of one variable changed by >25% when a variable was eliminated, then it was considered a confounder and put back into the multivariable model. Drugs included in the final multivariable model with a *P*-value of ≤0.05 were termed significant in this study.

Two separate multivariable logistic regression models were considered. The first multivariable model quantified the relationship between cases (oclacitinib treated dogs) and controls and overall drug use (Model 1). A second multivariable model quantified differences between cases and controls with regards to individual drug data, specifically different doses and mean number of courses per animal for an individual drug (Model 2). This was achieved by including the total number of courses per animal for total antibacterial, glucocorticoid and other drug use (including antihistamines and cyclosporin) for all animals. Due to the significant level of missing data (i.e., 38% in cases and 32% in controls) the length (i.e., duration in days) of antibacterial courses was not included in our models.

#### Models Adjusted for Type of Skin Condition and Presence of An Ear Condition

To determine if skin and ear conditions influenced antibacterial use in oclacitinib treated dogs (cases) and controls, Model 1 and Model 2 were divided into four sub models. Firstly, skin condition categories and changes in these categories were included in two sub models. Skin conditions were allocated to three categories; allergic dermatitis without secondary infection, allergic dermatitis with secondary superficial bacterial pyoderma and allergic dermatitis with secondary deep bacterial pyoderma (Supplementary Table 6). Changes were recorded if there were variations in skin condition category after initial consultations where oclacitinib was prescribed in cases and not prescribed in controls. Given the time-series nature of our database we constructed an explanatory variable that captures the dynamic nature without overfitting the model with temporal lags; as a result the proxy variable to measure skin condition changes in dogs was used to determine whether there was a general positive or negative response after treatment with oclacitinib.

The first sub model adjusted drug effects for baseline skin condition category (Model A) and the second sub model adjusted drug effects for changes in skin condition category for individuals diagnosed with a skin condition at the initial consultation (Model B). Individuals that experienced ear conditions instead of skin conditions were omitted from Model B. A further two sub models were created to determine whether the presence of ear conditions and changes in causative infectious agents influenced drug use in cases and controls. Ear condition was used as an overarching term that included all pruritic ears that could be both infected and uninfected. The ear condition variable was divided into seven categories. These included pruritic ears without the presence of an infectious agent, presence of cocci, presence of rods, presence of gram positive bacteria, presence of gram negative bacteria, a ruptured tympanic membrane and presence of *Malassezia pachydermatis* (Supplementary Table 6). The third sub model adjusted drug effects for the presence or absence of an ear condition (Model C) and the fourth model adjusted drug effects for the changes between infectious agents isolated from ears for individuals that experienced ear conditions (Model D).

Interaction terms between skin condition category and changes between skin condition categories and ear conditions and changes between infectious agents were also tested but due to the low number of events for some variables (Supplementary Tables 7, 8) we reported fixed effect logistic regression models only. All multivariable statistical analyses were performed using the statistical software Stata version 13.1.

## Results

### Dataset for Analysis

The search for clinical records resulted in a one to four ratio for dogs treated with oclacitinib (cases = 58) and dogs treated with other therapeutics (controls = 205). The distribution of sex, neuter status, age, and breed are displayed in Table 1. General practice consultations represented 93% of cases and 90% of controls. Dermatology specialist consultations accounted for 1% of visits for both groups. The remaining consultation types included internal medicine, surgical or hospitalized patients. The proportions of small, medium and large breeds were evenly distributed in cases and controls. There were more small breeds in both groups. Small breeds accounted for half of the cases and controls, where medium and large breeds each accounted for 21–25% of cases and controls (Table 1). Information on the length of courses in days for each antibiotic is available in Supplementary Table 9. The majority of dogs were treated with antibiotics empirically with only 16 (4 cases and 12 controls) having culture and susceptibility tests performed. Once test results were available dogs were treated with an appropriate antibiotic (i.e., one that for which the isolated bacteria was susceptible).

**Table 1:** Demographic data of dogs for cases and controls.

**Variables**	**Cases**	**Controls**
Total	58	205
**Sex**		
Male	28 (48%)	100 (49%)
Female	30 (52%)	105 (51%)
**Neuter status**		
Neutered	48 (83%)	150 (73%)
Entire	10 (17%)	55 (27%)
**Age (years)**		
1–4	22 (38%)	77 (38%)
>4–8	21 (36%)	77 (38%)
>8	15 (26%)	51 (24%)
**Breed**		
Small	29 (50%)	110 (54%)
Medium	14 (24%)	44 (21%)
Large	15 (25%)	51 (24%)

*Supplementary Tables 4a,b summarize the distribution of breeds in the three groups based on (19)*.

### Descriptive Results

#### Distribution of Antibacterial Use in Dogs Treated With Oclacitinib (Cases) vs. Dogs Not Treated With Oclacitinib (Controls)

**Figure 1:**
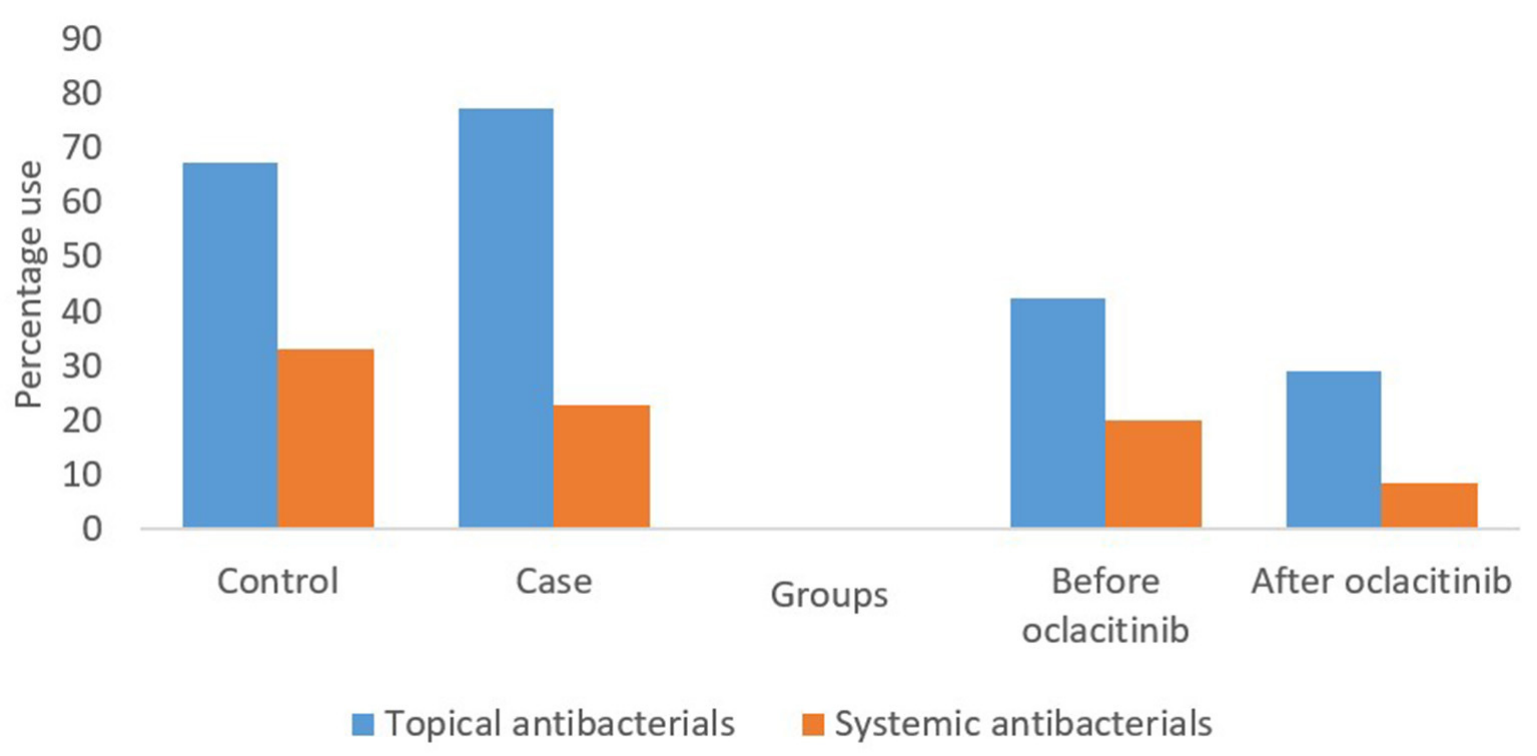
Systemic and topical antibacterial use in 58 cases and 205 controls with allergic dermatitis for all consults included in the study. The first two sets of bars represent the percentages of topical and systemic antibacterial courses prescribed in cases after oclacitinib use and controls. The final two sets of bars represent the percentages of topical and systemic antibacterial prescribed in cases before and after oclacitinib.

Total antibacterial use is shown in Figure 1. Both dogs treated with oclacitinib (cases) and dogs not treated with oclacitinib (controls) were prescribed a higher proportion of topical than systemic antibacterials. Cephalexin courses constituted 94% (17/18) of systemic antibacterial courses in the cases after initial oclacitinib use and 72% (125/173) in the controls. Amoxycillin clavulanic acid comprised 6% (1/18) of systemic antibacterial courses in the cases after initial oclacitinib use and 28% (48/173) in the controls. There were no significant differences in proportional use of systemic antibacterials or topical antibacterials in the cases and controls (*P* = 0.98).

**Figure 2:**
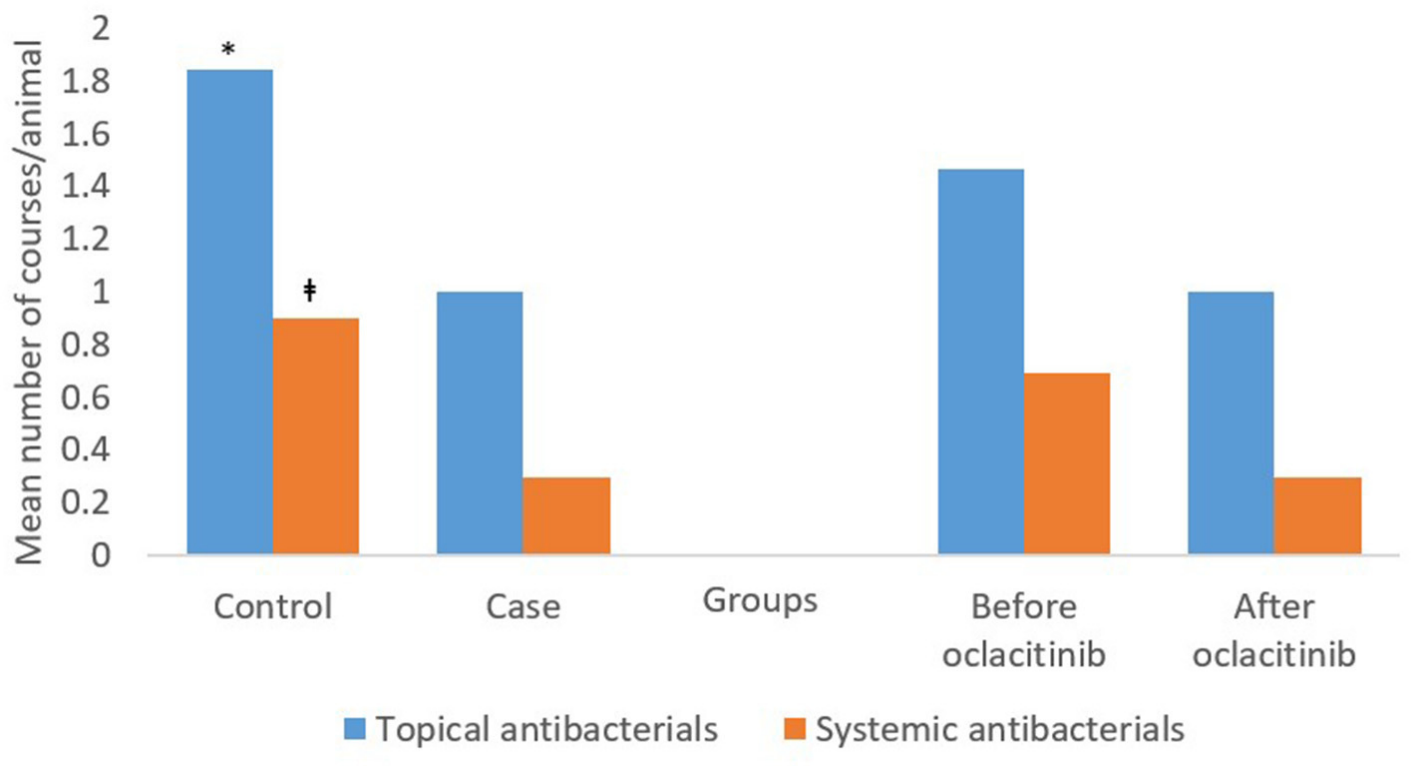
The mean topical and systemic antibacterial courses per animal in 58 cases and 205 controls, and before and after initial oclacitinib use in 58 cases for all consults included in the study. *P = 0.019 vs. cases; ^‡^*P* < 0.001 vs. cases. The first two sets of bars represent the percentages of topical and systemic antibacterial courses prescribed in cases after oclacitinib use and controls. The final two sets of bars represent the percentages of topical and systemic antibacterial prescribed in cases before and after oclacitinib.

There were twice as many topical and systemic antibacterial courses in total per animal in controls [total: mean (95% CI) 2.7 (2.27–3.21)] than in cases [1.3 (0.93–1.66)] (*P* < 0.001) (Figure 2). The difference between the total number of topical (*P* = 0.019) or systemic (*P* < 0.001) antibacterial courses per animal in the cases vs. controls was also significant. Cases had a significantly (*P* = 0.016) lower total number of cephalexin courses [0.3 (0.15–0.44)] per animal than controls [0.6 (0.49–0.73)]. Cases also had a significantly (*P* = 0.002*)* lower total number of amoxycillin clavulanic acid courses per animal [0.01 (−0.02–0.05)] than controls [0.2 (0.16–0.31)] (Supplementary Figure 1).

Controls had higher mean numbers of courses of topical antibiotics such as chlorhexidine, salicylic acid, polymyxin B, neomycin, fusidic acid and ciprofloxacin (Supplementary Figure 3). It is important to note that polymixin B, enrofloxacin and ciprofloxacin are considered as Highest Priority Critically Important Antimicrobials (21). While cases had more courses of florfenicol (Supplementary Figure 3), actual use of this drug was extremely low in the study population. None of the differences in topical treatments between cases and controls were statistically significant.

The mean number of topical or systemic courses per animal was lower in cases vs. controls for small, medium and large breeds (Supplementary Figures 5, 7, 9). In small breeds, this difference for cases vs. controls was significant for both topical (*P* = 0.016) and systemic (*P* < 0.001) antibacterials, however there was no significant difference between cases and controls in medium or large breeds.

No significant differences were identified in total antimicrobial use (*p* = 0.7094), total topical antimicrobial use (*p* = 0.5852), or total systemic antimicrobial use (*p* = 0.7509) between dogs that received different amounts of oclacitinib courses (one, two or three or more).

#### Distribution of Antibacterial Use Before vs. After Oclacitinib Use Within Cases

Topical antibacterials comprised a higher proportion of total antibacterials prescribed, both before and after oclacitinib use in cases (Figure 1). There were no significant differences in the proportional use of systemic or topical antibacterials (*P* = 0.201) before or after oclacitinib use. Sixty-one percent (27/44) of cephalexin courses were administered before initial oclacitinib use and 88% (7/8) of the amoxycillin clavulanic acid courses prescribed for cases were prescribed before initial oclacitinib use. There was no statistically significant differences between these proportions (*P* = 0.305).

The mean [95%CI] number of total antibacterial courses (systemic plus topical) per animal was higher before [2.2 (1.32–3)] than after [1.3 (0.93–1.66)] initial oclacitinib use. The difference between total number of topical or systemic antibacterial courses per animal before vs. after initial oclacitinib use was not significant) (Figure 2). After initial oclacitinib use, the total number of amoxycillin clavulanic acid (*P* = 0.048) and cephalexin courses (*P* = 0.336) per animal decreased (Supplementary Figure 2). While cases had higher mean numbers of courses for salicylic acid, neomycin, polymyxin B, florfenicol, enrofloxacin, and ciprofloxacin before (vs. after) oclacitinib use (Supplementary Figure 4), none of the differences were statistically significant.

Supplementary Figures 6, 8, 10 display the mean number of courses per breed group before and after initial oclacitinib use in the 58 cases. Both topical and systemic antibacterial use was more numerous in cases before initial oclacitinib use in small and medium breeds. Little to no difference was observed in large breeds. There was a significant difference in the number of systemic antibacterials used by small breeds before and after oclacitinib (*P* = 0.002). There were no significant differences in systemic or topical antibacterial use in medium or large breeds before or after oclacitinib use.

### Multivariable Logistic Regression Models

#### Overall Antibacterial Usage in Dogs Treated With Oclacitinib (Cases) vs. Dogs Not Treated With Oclacitinib (Controls)

Univariable analyses revealed that the total number of courses of antibacterials, glucocorticoids or “other” drug use had a significant effect on the outcome of being a case (Table 2). The odds of antibacterial use overall was lower in dogs treated with oclacitinib than controls [OR: 0.72 (0.59–0.88); *P* = 0.001]. Similarly, the odds of topical [OR: 0.78 (0.63–0.96); *P* = 0.02] and systemic [OR: 0.4 (0.24–0.66); *P* < 0.001] antibacterial use was lower in cases than controls. The odds of glucocorticoid use was also lower in cases than controls for overall [OR: 0.46 (0.33–0.64); *P* < 0.001], topical [OR: 0.54 (0.37–0.78); *P* = 0.001], and systemic [OR: 0.3 (0.17–0.54); *P* < 0.001] glucocorticoid use. The odds of “other” treatments were also lower in cases vs. controls [OR: 0.4 (0.14–1.14); *P* = 0.086]. Initial logistic regression results suggested that the outcome of being a case was also significantly associated with the use of chlorhexidine, cephalexin, neomycin, polymyxin B, amoxycillin clavulanic acid, prednisolone acetate, hydrocortisone, systemic prednisolone and chlorpheniramine with the odds of the use of any of these agents being lower in cases than controls.

**Table 2:** The univariable results of associations between cases and controls with respect to signalment, drug therapy and, skin or ear conditions.

**Variable**	**Odds ratio (95% confidence interval)**	***P* value**	**Overall *P* value**
**Age (years)**			0.980
1–4	Reference		
>4–8	0.95 (0.49–1.88)	0.893	
>8	1.03 (0.49–2.17)	0.939	
**Sex**			
Male	Reference		
Female	1.02 (0.57–1.83)	0.946	
**Breed**^□^			0.870
Small	Reference		
Medium	1.21 (0.58–2.5)	0.612	
Large	1.12 (0.55–2.26)	0.761	
**Neuter**			
Neutered	Reference		
Entire	0.57 (0.27–1.2)	0.139	
**Type of skin condition category at baseline**			<0.001
Non-bacterial allergic dermatitis	Reference		
No skin condition	0.73 (0.61–0.89)	0.001	
Superficial pyoderma	0.84 (0.76–0.94)	0.001	
**Changes in skin condition category**			
No change	Reference		
Improvement in skin condition category	1.25 (1.13–1.4)	<0.001	
**Ear condition**			
No ear condition	Reference		
Ear condition present	0.65 (0.34 −1.23)	0.183	
**Changes between infectious agents present in ears**			
No change	Reference		
Change present	0.51 (0.17–1.53)	0.228	
**Total number of courses**			
All antibacterials	0.72 (0.59–0.88)	0.001	
Topical antibacterials	0.78 (0.63–0.96)	0.02	
Systemic antibacterials	0.4 (0.24–0.66)	<0.001	
All glucocorticoids	0.46 (0.33–0.64)	<0.001	
Topical glucocorticoids	0.54 (0.37–0.78)	0.001	
Systemic glucocorticoids	0.3 (0.17–0.54)	<0.001	
Other (antihistamines and cyclosporin)	0.4 (0.14–1.14)	0.086	
**Number of courses of antibacterials**			
Chlorhexidine unknown frequency	0.17 (0.02–1.26)	0.082	
Chlorhexidine^*^	0.2 (0.03–1.41)	0.106	
Cephalexin^▪^	0.64 (0.4–1.03)	0.069	
Neomycin^*^	0.32 (0.13–0.81)	0.016	
Polymyxin B^*^	0.68 (0.45–1.04)	0.077	
Amoxycillin clavulanic acid^•^	0.13 (0.02–0.89)	0.038	
**Number of courses of other drugs**			
Prednisolone acetate^*^	0.7 (0.47–1.04)	0.075	
Hydrocortisone^*^	0.32 (0.13–0.81)	0.016	
Prednisolone^◦^	0.26 (0.11–0.65)	0.004	
Prednisolone ♢	2.05 (0.74–5.73)	0.169	
Chlorpheniramine	0.22 (0.03–1.44)	0.114	

* Topical antibacterial high frequency ≥ once per week ♢ Systemic tapering dose 1.1−4 mg/kg/day

◦ Systemic tapering dose 0.5–1 mg/kg/day

• *Systemic dose 12.5–25 mg/kg every 12 h*.

▪ Systemic dose 15–30 mg/kg every 12 h

□ *Breed categories defined in Supplementary Tables 4a,b*.

#### Effect of Skin and Ear Conditions in Dogs Treated With Oclacitinib (Cases) vs. Dogs Not Treated With Oclacitinib (Controls)

Our univariable analyses indicate that after initial oclacitinib use, cases had lower odds of having no skin conditions [OR: 0.73 (0.61–0.89); *P* = 0.001], or superficial pyoderma [OR: 0.84 (0.76–0.94); *P* = 0.001] at initial consultation, compared to controls (Table 2). In addition, cases had significantly higher odds of experiencing improvement in skin condition category vs. controls [OR: 1.25 (1.13–1.4); *P* < 0.001]. The majority of this improvement was dogs categorized with superficial pyoderma in the initial consult being categorized as a non-bacterial dermatitis due to allergic conditions at a subsequent consultation. Using this classification of change (changes earlier for models adjusted for type of skin condition and presence of ear condition), 48% of cases' skin conditions improved, 50% stayed the same and 2% worsened (Supplementary Table 5).

#### Effect of Baseline Skin Condition and Changes in Condition on Overall Antibacterial Usage in Dogs Treated With Oclacitinib (Cases) vs. Dogs Not Treated With Oclacitinib (Controls)

After accounting for age, sex, breed, neuter status and categorization of skin condition at baseline, the results of Model 1A determined that the odds of systemic antibacterial use [OR: 0.4 (0.18–0.92); *P* = 0.031], systemic glucocorticoid use [OR: 0.29 (0.15–0.56); *P* < 0.001] and use of other drugs (antihistamines and cyclosporin) [OR: 0.32 (0.1–0.97); *P* = 0.044] was lower in cases than in controls. After adjusting for baseline skin condition category and changes in skin condition category, the results in Model 1B were similar [odds of systemic antibacterial [OR: 0.29 (0.12–0.71); *P* = 0.007], glucocorticoid [OR: 0.29 (0.15–0.57); *P* < 0.001] and other drug [OR: 0.3 (0.1–0.93); *P* = 0.037] use was lower in cases]. Cases had higher odds of experiencing improvements in skin condition category vs. controls [OR: 7.89 (3.26–19.13); *P* < 0.001] (Table 3).

**Table 3:** Multivariable results displaying the association between case and controls with respect to overall drug use.

**Multivariable**	**Model 1A**			**Model 1B**		
**Variables**	**Odds ratio (95% confidence interval)**	***P-*value**	**Overall *P*-value**	**Odds ratio (95% confidence interval)**	***P*-value**	**Overall *P*-value**
**Age (years)**			0.93			0.96
1–4	Reference			Reference		
>4–8	1.08 (0.48–2.4)	0.857		1.13 (0.47–2.7)	0.78	
>8	0.91 (0.38–2.18)	0.828		1.09 (0.42–2.86)	0.862	
**Sex**						
Male	Reference			Reference		
Female	0.88 (0.44–1.76)	0.713		0.74 (0.34–1.6)	0.444	
**Breed**^□^			0.443			0.569
Small	Reference			Reference		
Medium	1.73 (0.73–4.09)	0.214		1.65 (0.63–4.31)	0.306	
Large	1.34 (0.58–3.08)	0.491		1.31 (0.53–3.21)	0.56	
**Neuter**						
Neutered	Reference			Reference		
Entire	0.44 (0.19–1.04)	0.061		0.41 (0.17–1.03)	0.059	
**Type of skin condition category at baseline**						
Non-bacterial allergic dermatitis	Reference			Reference		
Pyoderma (Superficial and deep)	0.89 (0.31–2.57)	0.835		0.83 (0.28–2.48)	0.746	
**Changes in skin condition category**						
No change				Reference		
Improvement in skin condition category				7.89 (3.26–19.13)	<0.001	
**Number of total drug courses**						
Systemic antibacterials	0.40 (0.18–0.92)	0.031		0.29 (0.12–0.71)	0.007	
Systemic glucocorticoids	0.29 (0.15–0.56)	<0.001		0.29 (0.15–0.57)	<0.001	
Total other (antihistamines and cyclosporin)	0.32 (0.1–0.97)	0.044		0.30 (0.1–0.93)	0.037	

*In Model A effects are adjusted for baseline skin condition category and in Model B effects are further adjusted for changes in skin condition. Variables with a P < 0.05 were considered statistically significant*.

□ *Breed categories defined in Supplementary Tables 4a,b*.

#### Effect of Individual Antibacterial Class and Glucocorticoid Usage Patterns in Dogs Treated With Oclacitinib (Cases) vs. Dogs Not Treated With Oclacitinib (Controls)

After accounting for age, sex, breed, neuter status and type of skin condition at baseline, the results of Model 2A indicate that the odds of topical neomycin use [OR: 0.26 (0.09–0.77); *P* = 0.015], amoxycillin clavulanic acid use [12.5–25 mg/kg every 12 h; OR: 0.12 (0.01–1); *P* = 0.050] and systemic prednisolone use [at a tapering dose of 0.5–1 mg/kg/day; OR: 0.23 (0.09–0.63); *P* = 0.004] were lower in cases compared to controls. After adjusting for baseline skin condition category and changes in skin condition category, the results in Model 2B were also similar (odds of use lower in cases vs. controls) for these doses of amoxycillin clavulanic acid [OR: 0.08 (0.01–0.71); *P* = 0.024] and prednisolone [OR: 0.26 (0.09–0.71); *P* = 0.009]. Neomycin was no longer retained as significant. Cases had much higher odds of experiencing improvements in skin condition category than controls [OR: 5.77 (2.51–13.28); *P* < 0.001] (Table 4).

**Table 4:** Multivariable results displaying the association between cases and controls with respect to specific drug types.

**Multivariable**	**Model 2A**			**Model 2B**		
**Variables**	**Odds ratio (95% confidence interval)**	***P*-value**	**Overall *P*-value**	**Odds ratio (95% confidence interval)**	***P*-value**	**Overall *P*-value**
**Age (years)**			0.907			0.985
1–4	Reference			Reference		
>4–8	1.2 (0.54–2.68)	0.659		1.07 (0.45–2.52)	0.885	
>8	1.08 (0.45–2.57)	0.86		1.08 (0.42–2.73)	0.878	
**Sex**						
Male	Reference			Reference		
Female	1.05 (0.52–2.09)	0.894		0.85 (0.4–1.81)	0.675	
**Breed**^□^			0.457			0.664
Small	Reference			Reference		
Medium	1.73 (0.7–4.26)	0.231		1.54 (0.58–4.13)	0.388	
Large	1.4 (0.6–3.29)	0.438		1.3 (0.52–3.25)	0.58	
**Neuter**						
Neutered	Reference			Reference		
Entire	0.48 (0.2–1.15)	0.100		0.42 (0.16–1.08)	0.073	
**Type of skin condition category at baseline**						
Non-bacterial allergic dermatitis	Reference			Reference		
Pyoderma (Superficial and deep)	0.56 (0.21–1.51)	0.253		0.51 (0.19–1.42)	0.20	
**Changes in skin condition category**						
No change				Reference		
Improvement in skin condition category				5.77 (2.51–13.28)	<0.001	
**Number of total drug courses**						
Neomycin^*^	0.26 (0.09–0.77)	0.015				
Amoxycillin clavulanic acid^•^	0.12 (0.01–1)	0.05		0.08 (0.01–0.71)	0.024	
Prednisolone °	0.23 (0.09–0.63)	0.004		0.26 (0.09–0.71)	0.009	

*In Model A effects are adjusted for baseline skin condition category and in Model B effects are further adjusted for changes in skin condition. Variables with a P < 0.05 were considered statistically significant*.

* Topical antibacterial high frequency ≥ once per week

• Systemic dose 12.5–25 mg/kg every 12 h ° Systemic tapering dose 0.5–1 mg/kg/day

□ *Breed categories defined in Supplementary Tables 4a,b*.

#### Effect of Ear Condition and Infectious Agents on Overall Antibacterial Usage in Dogs Treated With Oclacitinib (Cases) vs. Dogs Not Treated With Oclacitinib (Controls)

After accounting for age, sex, breed, neuter status and presence of an ear condition, the results of Model 1C showed the odds of systemic antibacterial [OR: 0.44 (0.25–0.77); *P* = 0.004] and systemic glucocorticoid [OR: 0.31 (0.16–0.6); *P* < 0.001] use were lower in cases than controls (Table 5). After adjusting for the presence of an ear condition and the changes in causative infectious agents, the results in Model 1D revealed no significant association between cases and drug therapies.

**Table 5:** Multivariable results of the association between drug cases and controls with respect to overall drug use.

**Multivariable**	**Model 1C**			**Model 1D**		
**Variables**	**Odds ratio (95% confidence interval)**	***P*-value**	**Overall *P*-value**	**Odds ratio (95% confidence interval)**	***P*-value**	**Overall *P*-value**
**Age (years)**			0.843			0.684
1–4	Reference			Reference		
>4–8	1.19 (0.55–2.58)	0.663		1.19 (0.28–5.02)	0.81	
>8	0.94 (0.4–2.17)	0.877		0.49 (0.06–3.68)	0.484	
**Sex**						
Male	Reference			Reference		
Female	0.97 (0.5–1.91)	0.938		0.92 (0.25–3.48)	0.908	
**Breed**^□^			0.545			0.499
Small	Reference			Reference		
Medium	1.58 (0.7–3.58)	0.275		1.60 (0.27–9.49)	0.604	
Large	1.23 (0.56–2.71)	0.613		2.26 (0.58–8.86)	0.243	
**Neuter**						
Neutered	Reference			Reference		
Entire	0.46 (0.2–1.05)	0.066		0.84 (0.17–4.09)	0.833	
**Ear condition**						
No ear condition	Reference					
Ear condition present	0.86(0.35–2.12)	0.743				
**Changes between infectious agents present in ears**						
No change				Reference		
Change present				1.09 (0.24–4.84)	0.914	
**Number of total drug courses**						
Systemic antibacterials	0.44 (0.25–0.77)	0.004				
Systemic glucocorticoids	0.31 (0.16–0.6)	<0.001				

*In Model C drug effects were adjusted for the presence (or absence) of an ear condition and Model D drug effects were adjusted for changes between infectious agents isolated from ears. Variables with a P < 0.05 were significantly associated*.

□ *Breed categories defined in Supplementary Tables 4a,b*.

#### Effect of an Ear Condition and Infectious Agents Individual Antibacterial Class Usage Patterns in Dogs Treated With Oclacitinib (Cases) vs. Dogs Not Treated With Oclacitinib (Controls)

After accounting for age, sex, breed, neuter status and presence of an ear condition, the results of Model 2C determined that the odds of neomycin use at high frequencies [OR: 0.3 (0.1–0.89); *P* = 0.029], amoxycillin clavulanic acid use at 12.5–25 mg/kg every 12 h [OR: 0.1 (0.01–0.83); *P* = 0.032] and systemic prednisolone use at a tapering dose of 0.5–1 mg/kg/day [OR: 0.25 (0.1–0.67); *P* = 0.006] were lower in cases compared to controls (Table 6). After adjusting for the presence of an ear condition and the changes in causative infectious agents, the results in Model 1D revealed that that the odds of neomycin use at high frequencies was lower in cases [OR: 0.07 (0.01–0.95); *P* = 0.045]. Majority of antibacterial treatments for ear conditions included topical polymyxin B sulfate, chlorhexidine and salicylic acid. Polymyxin B suphate was prescribed in 47% of all consults (controls and cases after oclacitinib administration) that were for pruritic ears without infectious agents, and 52% of all consults for pruritic ears that had infectious agents present on an ear swab. Salicylic acid was prescribed in 20% of consults for pruritic ears without infectious agents and 11% of pruritic ears with infectious agents. Chlorhexidine was prescribed in 10% of pruritic ears without infectious agents and 6% of pruritic ears with infectious agents. The differences in use of these three antibacterials were not a significant results in the final model (*p* > 0.05).

**Table 6:** Multivariable results of the association between cases and controls with respect to specific drug use.

**Multivariable**	**Model 2C**			**Model 2D**		
**Variables**	**Odds ratio (95% confidence interval)**	***P*-value**	**Overall *P-*value**	**Odds ratio (95% confidence interval)**	***P*-value**	**Overall *P*-value**
**Age (years)**			0.748			0.207
1–4	Reference			Reference		
>4–8	1.33 (0.61–2.91)	0.471		1.47 (0.32–6.74)	0.619	
>8	1.04 (0.45–2.4)	0.929		0.17 (0.02–1.86)	0.147	
**Sex**						
Male	Reference			Reference		
Female	1.17 (0.6–2.28)	0.648		0.77 (0.18–3.2)	0.716	
**Breed**^□^			0.610			0.160
Small	Reference			Reference		
Medium	1.44 (0.61–3.38)	0.403		1.04 (0.14–7.47)	0.973	
Large	1.39 (0.61–3.16)	0.433		5.61 (0.9–35.02)	0.065	
**Neuter**						
Neutered	Reference			Reference		
Entire	0.45 (0.19–1.07)	0.072		0.14 (0.02–1.25)	0.078	
**Ear condition**						
No ear condition	Reference					
Ear condition present	0.69 (0.27–1.76)	0.439				
**Changes between infectious agents present in ears**						
No change				Reference		
Change present				0.82 (0.17–4.02)	0.808	
**Number of total drug courses**						
Neomycin^*^	0.30 (0.1–0.89)	0.029		0.07 (0.01–0.95)	0.045	
Amoxycillin clavulanic acid^•^	0.10 (0.01–0.83)	0.032				
Prednisolone °	0.25 (0.1–0.67)	0.006				

*In Model C drug effects were adjusted for the presence (or absence) of an ear condition and Model D drug effects were adjusted for changes between infectious agents isolated from ears. Variables with a P < 0.05 were considered statistically significant*.

* Topical antibacterial high frequency ≥ once per week

• Systemic dose 12.5–25 mg/kg every 12 h ° Systemic tapering dose 0.5–1 mg/kg/day

□ *Breed categories defined in Supplementary Tables 4a,b*.

## Discussion

Antimicrobial resistance (AMR) is a public health emergency and antimicrobial stewardship in veterinary practice can help reduce the pressure on the development of AMR (22). One strategy to combat the emergence of AMR in veterinary practice is to reduce, when possible, the amount of antibacterials used. Historically, empirical systemic antibacterial therapy has been the standard treatment protocol for canine skin infections associated with allergic skin disease and our data are an example of this approach to the management of both cases and controls. However, with the emergence of antibacterial resistance, topical antibacterial therapies are considered an important monotherapy or adjunct therapy (23). Therapeutic agents with potential to contribute to such reduction in use of antibacterials are important tools in the improvement of antimicrobial stewardship in veterinary medicine. Oclacitinib which is used to treat allergic dermatitis, including atopic dermatitis acts by targeting specific pathways involved in itch and inflammation. Alleviating pruritus can interrupt the itch cycle and allow for skin healing, thus aiding reduction of the chronic inflammatory changes and secondary infections (10). This study, which aimed to quantify topical and systemic antibacterial use, corticosteroid use and the effect on skin categories in client-owned dogs with allergic dermatitis, including atopic dermatitis, treated with oclacitinib compared to dogs treated with other common therapies, showed that cases treated with oclacitinib had an overall reduction in their percentage usage of systemic and topical antibacterials and an improvement in their skin conditions.

In this study, cephalexin and amoxycillin clavulanic acid were the primary systemic antibacterials used in both cases and controls. Both agents are recommended as first-line antibacterials to empirically treat superficial canine pyoderma (17, 24). Our results revealed that dogs with allergic skin disease treated with oclacitinib had significantly (*P* < 0.05) lower odds of needing systemic antibacterials than dogs treated with other therapies. Further analysis revealed that the odds were lower specifically for amoxycillin clavulanic acid usage at 12.5–25 mg/kg every 12 h. This result remained significant after adjusting for age, sex, breed, neuter status, presence of ear condition, skin condition at initial consult and changes between skin condition categories. We also showed that before adjusting for demographic, drug therapy, skin condition or ear condition variables, the number of cephalexin courses per animal was significantly (*P* < 0.05) reduced in dogs treated with oclacitinib. However, after accounting for important confounders in the multivariable model, the number of cephalexin courses was not significantly different between cases and controls, which could be partly explained by the dominance of cephalexin prescriptions in both groups of dogs.

In our study topical therapies represented a larger proportion of total antibacterial courses than systemic therapies in dogs receiving oclacitinib and those treated with other therapies. Overall, our univariable results demonstrate that topical antibacterial use was reduced after oclacitinib use; however, after adjustment for age, sex, breed, neuter status, presence of ear condition, skin condition at initial consult and changes between skin condition categories in the multivariable models the only significant reduction in a specific antibacterial was in topical neomycin use at a frequency of more than once a week. The use of enrofloxacin, ciprofloxacin and Polymyxin B, which are Highest Priority Critically Important Antimicrobials, were not significantly reduced, highlighting the importance of antibiotic stewardship (21).

Glucocorticoids are another commonly used therapy to treat allergic dogs (10). Multivariable logistic regression analyses indicated that the odds of overall systemic glucocorticoid use were lower in dogs treated with oclacitinib, specifically systemic prednisolone usage at a tapering dose of 0.5–1 mg/kg/day. This result was retained after adjusting for age, sex, breed, neuter status, the presence of ear condition, skin conditions at baseline and changes between them. It has been suggested that the concomitant use of oral glucocorticoids with oclacitinib is discouraged due to immunosuppression concerns. Currently there are no published data demonstrating the long-term safety of concomitant use, our data suggest that veterinarians were probably inclined to avoid prednisolone use when treating with oclacitinib as only three dogs were treated with both drugs simultaneously. In contrast, twenty-four dogs from the oclacitinib treated group were prescribed 41 courses of prednisolone prior to initial oclacitinib use. The efficacy of both drugs in treating pruritic conditions have been shown to be very similar (25). The indirect reduction in glucocorticoid use could be considered beneficial due to the adverse effects associated with long-term use (10). For example, long-term glucocorticoid use in dogs has been associated with an increased risk of developing a urinary tract infection (26) and one study found urinary tract infections in 39% of dogs receiving long-term corticosteroid therapy for chronic skin diseases (27).

In addition, the results of our study also suggested that dogs treated with oclacitinib were significantly more likely to experience improvements in their skin condition categories compared to dogs treated with other therapies, which is an additional benefit of using this treatment approach. A recent study suggested that oclacitinib use had positive effects on skin barrier parameters compared to prednisolone and other treatments, such as lower transepidermal water loss (TEWL) and increased hydration (28). TEWL is an indication of total cutaneous water loss and is often used to assess skin barrier activity, as an increase in TEWL has been associated with decreased skin integrity in atopic patients (29). Perhaps the hydration levels in the skin and/or return of skin microbiome to a normal flora is associated with an improvement in skin condition but these deserve further investigation.

There are a few limitations with our study primarily associated with the nature of the clinical record data used in our investigation which need to be addressed by further research. We attempted to record the average duration of an antibacterial course, but this information was not always available, especially with topical therapeutics. Therefore, we were unable to adjust for the length of antibacterial use in the final models. Information on why dogs in the control group did not receive oclacitinib was also not always available which may potentially confound some of the associations identified in this study. Details on diagnostic tests performed to confirm or rule out causes of itchy skin were not always available in the consultation notes. This was also true for diets and dietary changes in animals with food allergies as a definitive or differential diagnosis, where only eight dogs (one case and seven controls) experienced dietary changes. While we did not include the terms “otitis” and “pododermatitis”—given that these are not part of on label conditions—our search yielded some cases that had these conditions listed in clinical records. This indicates that oclacitinib is potentially being used with some success for a wider list of allergic dogs, the impact of which needs further investigation. In addition the effects noted are associations and better causal relationships could be ascertained using a prospective study design such as an intervention trial to confirm the associations noted in this study.

In conclusion, dogs with allergic skin disease treated with oclacitinib had a significantly reduced use of systemic and topical antibacterials and significantly higher odds of experiencing improvements in their skin condition category.

## Data Availability Statement

The raw data supporting the conclusions of this article will be made available by the authors, without undue reservation.

## Author Contributions

HR conducted the study, completed the analysis, and drafted the manuscript. JG, EM, and RS were involved in the study design and finalization of the manuscript. All authors contributed to the article and approved the submitted version.

## Conflict of Interest

All authors report grants from Zoetis Australia, Pty Ltd, during the conduct of the study.
