# Supplementary material for: The Association Between the Use of Oclacitinib and Antibacterial Therapy in Dogs With Allergic Dermatitis: A Retrospective Case-Control Study
Source: Front Vet Sci. 2021 Feb 15;8:631443. doi: 10.3389/fvets.2021.631443 (PMC7928369; doi:10.3389/fvets.2021.631443)
Supplement: Supplementary file 1 [file Data_Sheet_1.docx]

**Supplementary Data**

**Table S1.** Search terms used to identify cases and controls in the database

| **Search terms and phrases** |
| --- |
| Dermatitis, food allergies, flea allergies, flea allergy dermatitis, hot spot, acute moist dermatitis contact allergic dermatitis, pruritus, itching, atopy, atopic, cutaneous adverse food reaction, food hypersensitivity reaction superficial pyoderma, deep pyoderma, allergic dermatitis, flare of allergic dermatitis or atopic dermatitis, atopic flare, sarcoptic mange, chronic inflammatory skin disease |
| Acute moist dermatitis (hot spot) secondary to allergic dermatitis |
| Suspected allergic dermatitis with secondary Staphylococcal pyoderma |
| Suspected allergic dermatitis with secondary yeast dermatitis |
| Acute flare of atopic dermatitis |
| Initial presentation of allergic dermatitis (no infection or parasites) |
| Suspected flea allergy undergoing flea control trial |
| Suspected food allergy undergoing diet trial |
| Allergy testing |
| Ongoing allergen-specific immunotherapy |
| Combinations of these words and/or plurals and abbreviations,  e.g. FAD – flea allergy dermatitis was also used. |

**Table S2.** The information collected for each case and control

| **Demographic information** | **Clinical history** |
| --- | --- |
| Age | Consult type, date and outcome |
| Sex  Male  Female | Clinical signs  Pruritus  Alopecia  Rash/urticaria  Lesions  Erythema  Dermatitis  Erosion  Lichenification  Discharge  Dry/flaky skin  Epidermal collarettes  Inflammation  Comedones/papules/pustules  Ulceration  Salivary staining  Hyperpigmentation  Self-trauma  Purulent discharge  Hypotrichosis  Excoriations |
| Neuter status | Diagnostic tests and results  Presence of ectoparasites  Skin scrapes  Flea combs  Swabs  Sticky tapes  Biopsy  Cultures |
| Breed | Diagnosis and treatment plans |
| GPS coordinates of home location | Drugs administered, drug dose, frequency and duration for treatment of skin conditions. Drugs were grouped as follows:  Antimicrobials  Corticoids  Apoquel (Oclacitinib given per label)  Other (cyclosporine, antihistamines)  Doses were not always available for topical treatments and so frequency of use was recorded instead. High frequency was more than once a week, low frequency was less than once a week. |

**Table S3.** The factors included in logistic regression models

| **Individual factors** | **Antimicrobial factors** | **Corticoid factors** | **‘Other’ drug factors** |
| --- | --- | --- | --- |
| Breed | Chlorhexidine topical: frequency (high, low, unknown), course per frequency | Prednisolone acetate topical: frequency (high, low, unknown), course per frequency | Chlorpheniramine (present or absent) |
| Sex | Salicylic acid topical: frequency (high, low, unknown), course per frequency | Hydrocortisone topical: frequency (high, low, unknown), course per frequency | Cyclosporine (present or absent) |
| Neuter status | Neomycin topical: frequency (high, low, unknown), course per frequency | Hydrocortisone aceponate topical: frequency (high, low, unknown), course per frequency |  |
| Age | Polymyxin B topical: frequency (high, low, unknown), course per frequency | Dexamethasone topical: frequency (high, low, unknown), course per frequency |  |
| Skin conditions   - Non-infectious dermatitis - Superficial pyoderma - Deep pyoderma | Enrofloxacin topical: frequency (high, low, unknown), course per frequency | Betamethasone topical: frequency (high, low, unknown), course per frequency |  |
| Ear conditions and presence of causal agents identified by ear swab   - Gram positive/negative bacteria - Rods or cocci - Malassezia - Ruptured/non ruptured tympanic membrane | Fusidic acid topical: frequency (high, low, unknown), course per frequency | Prednisolone systemic:  4 doses;  0.1 mg/kg/d–0.5mg/kg/d,  0.5 mg/kg/d–1 mg/kg/d,  1 mg/kg/d–4 mg/kg/d, unknown. Total courses, duration, tapering. |  |
|  | Florfenicol topical: (frequency (high, low, unknown), course per frequency total courses, duration per course) |  |  |
|  | Ciprofloxacin topical: frequency (high, low, unknown), course per frequency |  |  |
|  | Cephalexin systemic: 4 dose groups; <15 mg/kg twice daily, 15–30 mg/kg twice daily, >30 mg/kg twice daily, unknown. Total courses and duration per dose. |  |  |
|  | Amoxycillin clavulanic acid systemic: 4 dose groups; <12.5 mg/kg twice daily, 12.5–25 mg/kg twice daily, >25 mg/kg twice daily, unknown. Total courses and duration per dose. |  |  |
|  | Enrofloxacin systemic: dose (5–9 mg/kg), total courses, duration per course. |  |  |

**Table S4a.** The distribution of breeds in the three groups based on size for 58 cases

| **Small (29)** | **Proportion** | **Medium (14)** | **Proportion** | **Large (15)** | **Proportion** |
| --- | --- | --- | --- | --- | --- |
| Beagle | 3% | Australian shepherd | 7% | Boxer | 20% |
| Chihuahua | 14% | Border collie | 7% | French mastiff | 7% |
| Cocker Spaniel | 7% | Bull Arab | 14% | German shepherd | 27% |
| Jack Russell | 3% | Bull terrier | 14% | Labrador | 7% |
| Maltese | 14% | Australian cattle dog | 14% | Mastiff | 13% |
| Miniature Fox terrier | 3% | Dalmatian | 14% | Murray river curly coated retriever | 7% |
| Pug | 14% | English Pointer | 7% | Rhodesian ridgeback | 7% |
| Staffordshire Bull terrier | 28% | Hungarian vizsla | 7% | Smithfield | 7% |
| Toy poodle | 11% | Australian kelpie | 7% | Wolfhound | 7% |
| West highland terrier | 3% | Poodle | 7% |  |  |

Included pure and cross breeds.

**Table S4b.** The distribution of breeds in the three groups based on size for 205 controls

| **Small (110)** | **Proportion** | **Medium (44)** | **Proportion** | **Large (51)** | **Proportion** |
| --- | --- | --- | --- | --- | --- |
| Australian terrier | 1% | Belgian shepherd | 2% | Akita | 2% |
| Beagle | 4% | Border collie | 18% | Alaskan malamute | 2% |
| Bichon frise | 1% | Bull arab | 11% | American bandogge | 2% |
| Boston terrier | 1% | Bulldog | 7% | Boxer | 2% |
| Cavoodle | 1% | Bull terrier | 5% | Bull mastiff | 8% |
| Chihuahua | 5% | Catahoula leopard dog | 2% | Chesapeake Bay retriever | 2% |
| CKCS | 8% | Australian cattle dog | 11% | Dogue de Bordeaux | 2% |
| Corgi | 1% | Dalmatian | 5% | German shepherd | 25% |
| Dachshund | 3% | English pointer | 2% | Golden retriever | 2% |
| Fox terrier | 3% | Australian kelpie | 9% | Great dane | 2% |
| Jack Russell | 5% | Pit bull terrier | 5% | Greyhound | 2% |
| Lhasa apso | 2% | Poodle | 11% | Irish wolfhound | 2% |
| Maltese | 15% | Samoyed | 2% | Labrador | 27% |
| Miniature fox terrier | 7% | Shar pei | 2% | Maremma sheepdog | 2% |
| Pomeranian | 3% | Siberian Husky | 2% | Mastiff | 2% |
| Pug | 2% | Springer spaniel | 5% | Rhodesian ridgeback | 4% |
| Shih tzu | 7% |  |  | Rottweiler | 6% |
| Silky terrier | 3% |  |  | Schnauzer | 2% |
| Staffordshire bull terrier | 18% |  |  | Smithfield | 2% |
| Terrier | 5% |  |  |  |  |
| Tibetan spaniel | 3% |  |  |  |  |
| Toy poodle | 1% |  |  |  |  |
| West highland terrier | 1% |  |  |  |  |
| Whippet | 1% |  |  |  |  |
| Yorkshire terrier | 1% |  |  |  |  |

**Table S5.** The percentage distribution of skin condition categories, ear conditions and the changes between them

| Variable | Categories | Case (58) | Control (205) |
| --- | --- | --- | --- |
| Skin condition at initial consult (baseline skin condition) | Nothing | 0% | 10%* |
|  | Non-bacterial dermatitis | 74% | 46% |
|  | Superficial pyoderma | 26% | 44% |
| Change in skin condition category | No change | 50% | 77% |
|  | Change to improved skin condition category | 48% | 18% |
|  | Change to worse skin condition category | 2% | 5% |
| Ear condition | Absent | 72% | 63% |
|  | Present | 28% | 37% |
| Change between infectious agent identified on ear swab | No change | 93% | 87% |
|  | Change | 7% | 13% |

*The animals that didn’t present with skin conditions initially had an ear condition.

**Table S6.** The changes in skin condition categories and agents causing otitis externa in 58 cases and 205 controls

| **Changes form initial consult to final consult** | **Cases (58)** | **Controls (205)** |
| --- | --- | --- |
| **Skin changes** | | |
| no change | 50% | 77% |
| superficial pyoderma > non- bacterial dermatitis | 10% | 13% |
| non- bacterial dermatitis > resolved at recheck | 38% | 0% |
| superficial pyoderma > resolved at recheck | 0% | 3% |
| non- bacterial dermatitis > superficial pyoderma | 0% | 3% |
| superficial pyoderma > deep pyoderma | 2% | 1% |
| non- bacterial dermatitis > deep pyoderma | 0% | 1% |
| deep pyoderma > superficial pyoderma | 0% | 1% |
| deep pyoderma > non- bacterial dermatitis | 0% | 0% |
|  |  |  |
| **Ears changes** | | |
| None | 93% | 87% |
| yeast > non- bacterial irritation | 2% | 3% |
| cocci/rods > cocci | 2% | 0% |
| non- bacterial irritation > cocci/yeast | 2% | 0% |
| yeast > cocci | 2% | 0% |
| non- bacterial irritation > cocci/rods | 0% | 1% |
| cocci/yeast > cocci | 0% | 1% |
| cocci/yeast > non- bacterial irritation | 0% | 1% |
| yeast > cocci/yeast | 0% | 1% |
| non- bacterial irritation > cocci | 0% | 0% |
| non- bacterial irritation > yeast | 0% | 0% |
| cocci/rods > cocci/rods/yeast | 0% | 0% |
| cocci > non- bacterial irritation | 0% | 0% |
| non-bacterial irritation > rods | 0% | 0% |
| yeast > cocci/rods/yeast | 0% | 0% |
| cocci/yeast > yeast | 0% | 0% |
| cocci > cocci/yeast | 0% | 0% |
| rods > cocci/rods | 0% | 0% |

**Table S7.** The number of dogs that experienced difference skin conditions and changes in skin condition categories

|  | **Change in skin condition category** | | |
| --- | --- | --- | --- |
| **Skin condition category at initial consult** | No change | Change | Total |
|  |  |  |  |
| No skin condition | 20 | 0 | 20 |
| Non-bacterial dermatitis | 104 | 34 | 138 |
| Superficial pyoderma | 63 | 42 | 105 |
|  |  |  |  |
| Total | 187 | 76 | 263 |

**Table S8.** The number of dogs that experienced an ear condition and changes in infectious agents

|  | **Change in skin condition category** | | |
| --- | --- | --- | --- |
| **Skin condition category at initial consult** | No change | Change | Total |
|  |  |  |  |
| No ear condition | 171 | 0 | 171 |
| Ear condition present | 62 | 30 | 92 |
|  |  |  |  |
| Total | 233 | 30 | 263 |

**Table S9.** The median, minimum and maximum lengths of courses in days per antibiotic used by all dogs in the study. Antibiotics with 3 or more total courses are included.

| **Antibiotic** | **Total number of courses** | **Median length of course** | **Minimum length of course** | **Maximum length of course** |
| --- | --- | --- | --- | --- |
| Cephalexin | 117 | 14 | 3 | 56 |
| Polymixin B Sulphate | 100 | 10 | 5 | 21 |
| Amoxycillin-clavulanic acid (Per Os) | 92 | 11 | 1 | 30 |
| Neomycin | 61 | 10 | 5 | 14 |
| Cephazolin | 17 | 1 | 1 | 4 |
| Metronidazole | 10 | 7 | 2 | 28 |
| Gentamicin | 7 | 7 | 5 | 7 |
| Doxycycline | 6 | 14 | 14 | 14 |
| Ciprofloxacin | 6 | 7 | 1 | 14 |
| Enrofloxacin (Per Os) | 5 | 7 | 5 | 42 |
| Enrofloxacin (Topical) | 5 | 7 | 1 | 50 |
| Fusidic acid | 5 | 9 | 7 | 10 |
| Tricin | 4 | 10 | 5 | 14 |
| Fusidic acid | 3 | 7 | 7 | 21 |

**Figure legends**

**Figure S1.** The mean number of systemic antimicrobial courses per animal for cephalexin and amoxycillin clavulanic acid (AMC) in cases after initial oclacitinib use and controls after initial treatment of allergic skin condition.

**P* = 0.016 and ǂP=0.002 for cases vs controls

**Figure S2.** The mean number of systemic antimicrobial courses per animal for cephalexin and amoxycillin clavulanic acid (AMC) before and after initial oclacitinib use in the 58 cases.

**P* = 0.025 before vs. after initial oclacitinib use.

**Figure S3.** The mean number of courses per animal for topical antimicrobials in cases after initial oclacitinib use and controls after initial treatment of allergic skin condition.

**Figure S4.** The mean number of courses per animal for topical antimicrobials before and after initial oclacitinib use in the 58 cases.

**Figure S5.** The mean number of courses per animal for total topical and total systemic antimicrobial use in small breeds (29 cases) after initial oclacitinib use and (110 controls) after initial treatment of allergic skin condition.

**P* = 0.030 and ǂ*P* = 0.004 for cases vs. controls

**Figure S6.** The mean number of courses per animal for total topical and total systemic antimicrobial use in small breeds (29 cases) before and after initial oclacitinib use.

**P* = 0.00198

**Figure S7.** The mean number of courses per animal for total topical and total systemic antimicrobial use in medium breeds (14 cases) after initial oclacitinib use and (44 controls) after initial treatment of allergic skin condition.

**Figure S8.** The mean number of courses per animal for total topical and total systemic antimicrobial use in medium breeds (14 cases) before and after initial oclacitinib use.

**Figure S9.** The mean number of courses per animal for total topical and total systemic antimicrobial use in large breeds (15 cases) after initial oclacitinib use and (51 controls) after initial treatment of allergic skin condition.

**Figure S10.** The mean number of courses per animal for total topical and total systemic antimicrobial use in large breeds (15 cases) before and after initial oclacitinib use.
