# Supplementary figures and images for: The Association Between the Use of Oclacitinib and Antibacterial Therapy in Dogs With Allergic Dermatitis: A Retrospective Case-Control Study
Source: Front Vet Sci. 2021 Feb 15;8:631443. doi: 10.3389/fvets.2021.631443 (PMC7928369; doi:10.3389/fvets.2021.631443)

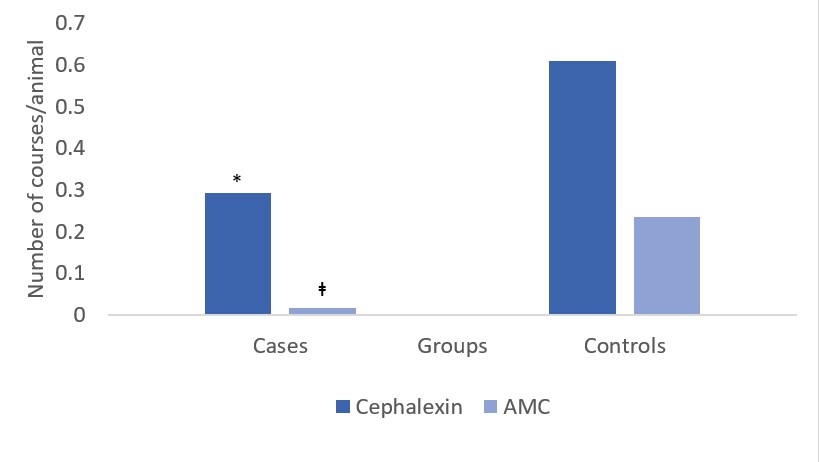

Supplement: Supplementary file 2 [file Image_1.JPEG]

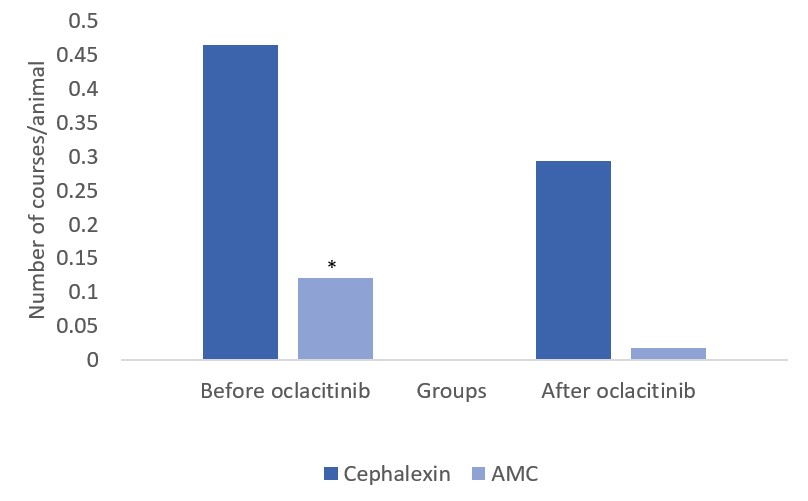

Supplement: Supplementary file 3 [file Image_2.JPEG]

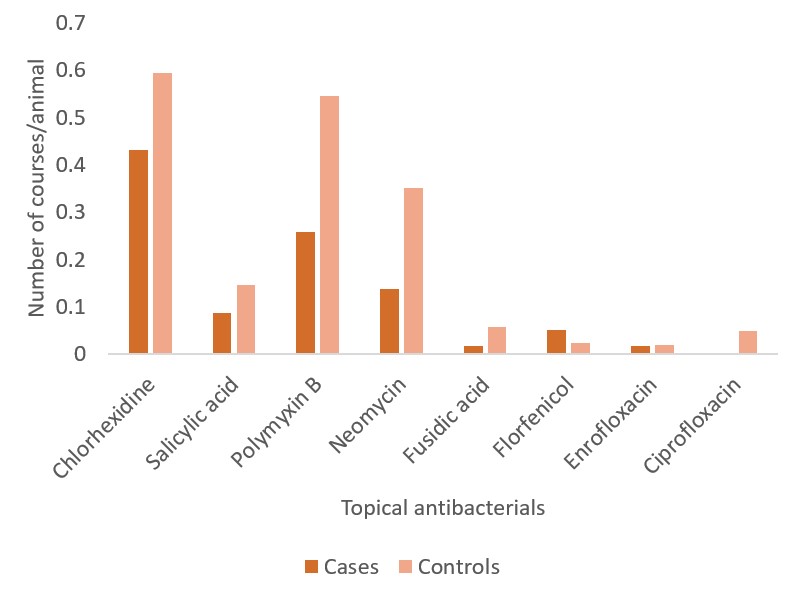

Supplement: Supplementary file 4 [file Image_3.JPEG]

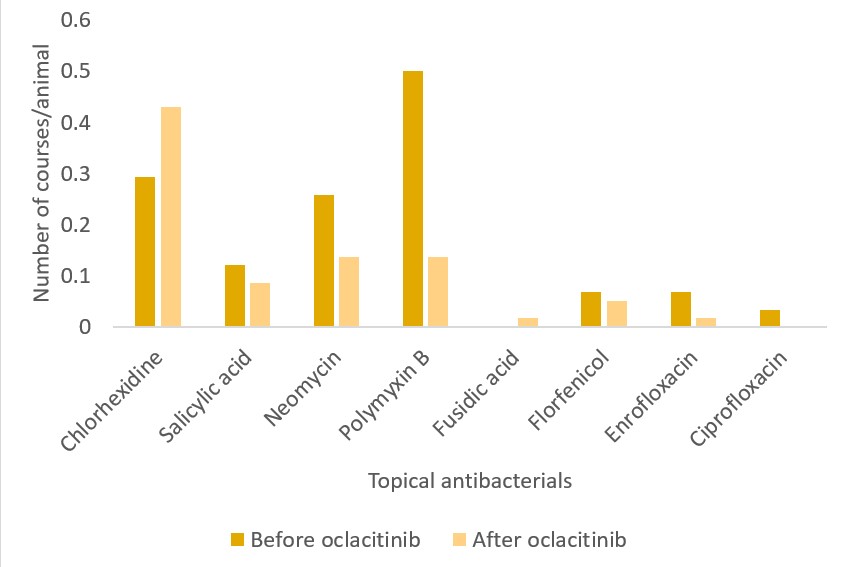

Supplement: Supplementary file 5 [file Image_4.JPEG]

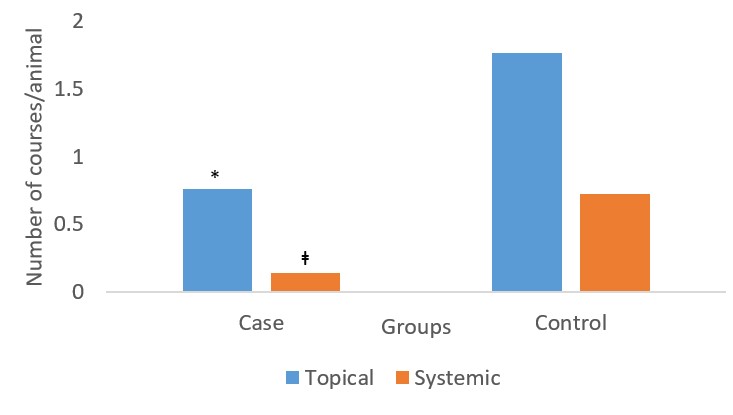

Supplement: Supplementary file 6 [file Image_5.JPEG]

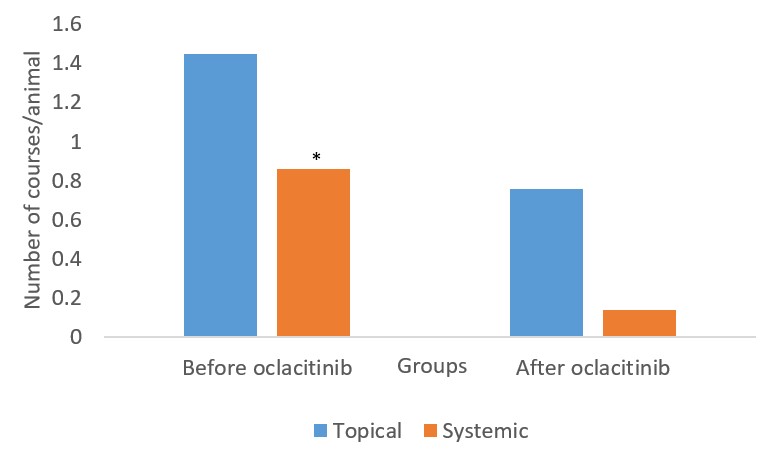

Supplement: Supplementary file 7 [file Image_6.JPEG]

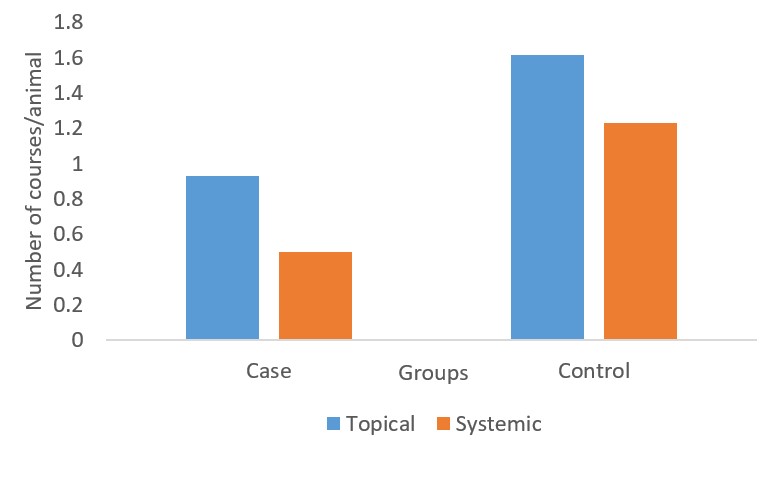

Supplement: Supplementary file 8 [file Image_7.JPEG]

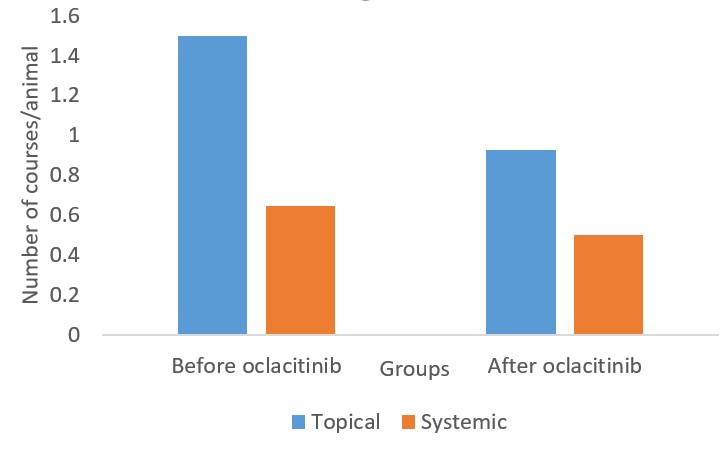

Supplement: Supplementary file 9 [file Image_8.JPEG]

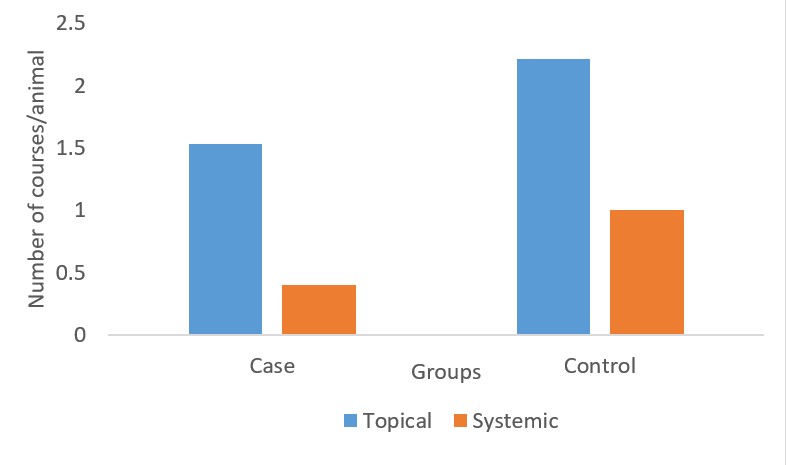

Supplement: Supplementary file 10 [file Image_9.JPEG]

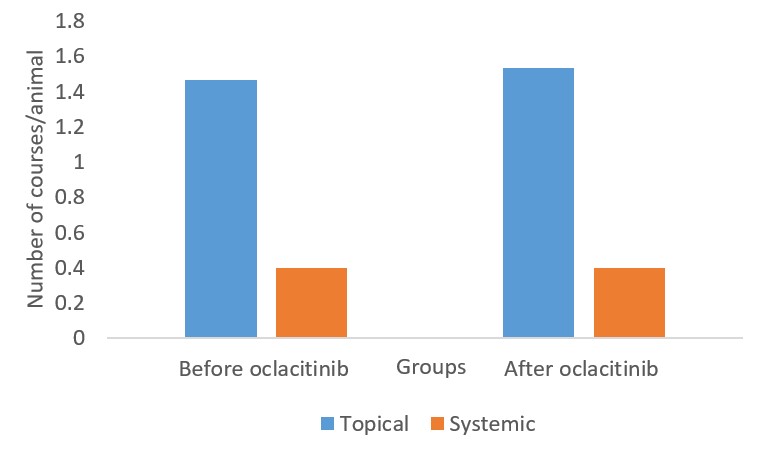

Supplement: Supplementary file 11 [file Image_10.JPEG]

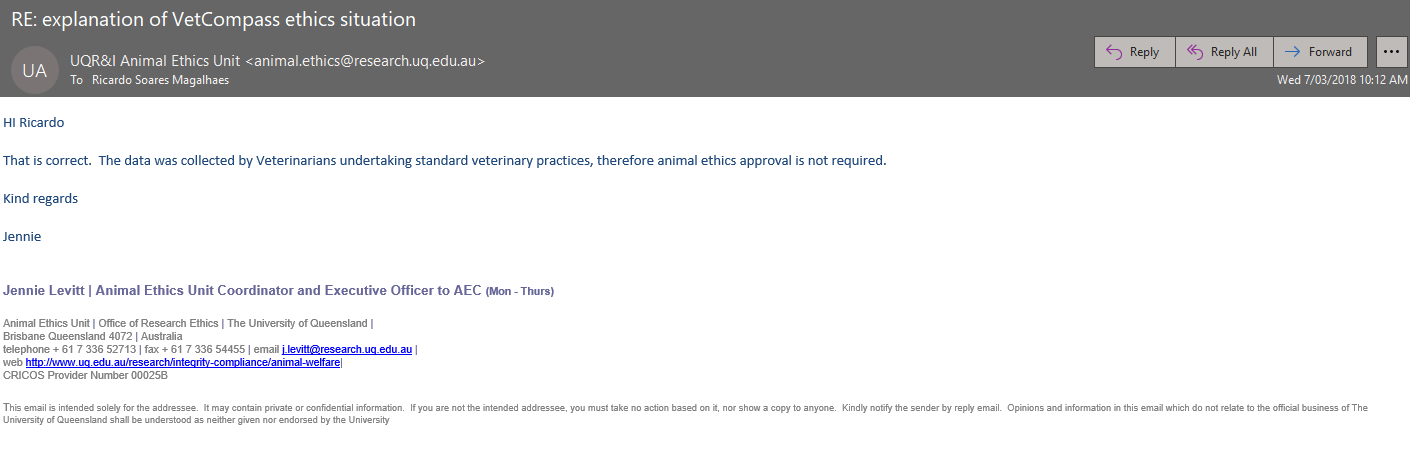

Supplement: Supplementary file 12 [file Image_11.PNG]
